# Maternal-fetal dialogue and immunological programming: from fetal development to long-term health outcomes

**DOI:** 10.3389/fonc.2026.1951828

**Published:** 2026-09-18

**Authors:** Marina S. Folguieri, Juliana Baboghlian, Sepideh Razi, Lucas Assoni, Leonardo O. Reis

**Affiliations:** 1 UroScience, School of Medical Sciences, University of Campinas (UNICAMP), São Paulo, Brazil; 2 INCT-UroGen, National Institute of Science, Technology and Innovation in Genitourinary Cancer, São Paulo, Brazil; 3 ImmunOncology, Pontifical Catholic University of Campinas (PUC-Campinas), São Paulo, Brazil

**Keywords:** epigenetic regulation, fetal immunological programming, hematopoiesis, immune-mediated diseases, immunogenomics, materno-fetal interface, precision immunology, trained immunity

## Abstract

The prenatal period is a critical phase of high cellular plasticity, during which the immune system develops while maintaining essential maternal-fetal tolerance. This review examines the ontogeny of the hematopoietic system and the sequential maturation of immune components, as well as the mechanisms through which maternal inflammatory and microbial signals act as epigenetic cues that can program the offspring’s long-term immunological resilience. Rather than merely a precursor to the adult immune system, the fetal and neonatal immune configuration is a highly specialized state, functionally balanced to tolerate maternal microchimerism while preparing for postnatal environmental exposure. Ultimately, early-life programming, influenced by a wide range of factors, including maternal nutrition and environmental conditions, determines future susceptibility to infectious, inflammatory, and neoplastic diseases, offering a vital framework for developing new immunogenomic biomarkers and precision immunotherapies in adulthood.

## 1 Introduction

Adaptation to the environment occurs through three mechanisms: physiological responses, which are rapid and reversible (seconds to hours); acclimatization, involving stable yet reversible physiological adjustments to prolonged environmental changes (days to months); and developmental plasticity, which operates during critical periods of development, shaping biological processes and generating adaptive phenotypes for anticipated future environments (1). Unlike the other mechanisms, developmental programming tends to produce long-lasting changes that may become maladaptive when the environment encountered later in life does not match the conditions anticipated during development. Although substantial epidemiological evidence supports this concept, the biological mechanisms responsible for establishing and maintaining these adaptations remain incompletely understood (2).

Within this framework, the immune system represents a particularly critical target for developmental programming. Given its role in balancing host protection with self-tolerance, the fetal immune system must integrate maternal environmental signals to calibrate its functional set-points for postnatal life. However, how these maternal-fetal interactions specifically dictate the trajectory of immunological maturation and susceptibility to chronic disease remains a fundamental question in current pathophysiology (2, 3).

This review examines the ontogeny of the fetal immune system and discusses the mechanisms through which maternal environmental inputs may shape fetal immune development and contribute to long-term immunological programming. By synthesizing these developmental pathways, we argue that early-life programming represents an under-explored dimension of inter-individual variability in immune responses, potentially serving as a source of novel immunogenomic biomarkers that dictate susceptibility to chronic disease and responsiveness to precision immunotherapy in adulthood.

### 1.1 The immunological coordination of gestation and fetal ontogeny

Contemporary gestational immunology has shifted from passive tolerance to recognizing the immune system as an active coordinator of the dynamic physiological changes required for implantation, placentation, and fetal growth. Rather than a monolithic entity, the maternal immune system undergoes continuous modulation through interactions with fetal signaling and environmental challenges, relying on high plasticity to adapt to the shifting biological demands and critical transitions of each gestational stage (4, 5).

Historically, the fetus has been regarded as a semi-allogeneic graft that requires finely regulated maternal immune tolerance to avoid rejection. It is now recognized that pregnancy involves dynamic bidirectional immunological crosstalk between the mother and fetus, mediated in part by maternal-fetal microchimerism. This bidirectional cellular trafficking transforms the placenta into a continuous signaling interface, exposing the developing fetal hematopoietic system to maternal biological information and microbial metabolites. This early contact serves as a priming stimulus that induces chromatin remodeling and epigenetic alterations within fetal progenitors, establishing the foundation for trained immunity and calibrating postnatal innate reactivity (6).

Even prior to this exchange, successful implantation and placentation necessitate a molecular dialogue between the blastocyst and the endometrium, shifting the endometrial microenvironment toward a receptive state. This endometrial immunological receptivity is currently considered a deterministic factor in reproductive success, underscoring that precise immunological regulation is the foundation for healthy fetal development (7).

For successful implantation and placentation, an initial invasive pro-inflammatory stage is required to breach the endometrial epithelium and facilitate trophoblast invasion, a process in which the immune system essentially “breaches” the endometrium to allow placental anchoring (8). This phase is followed by an anti-inflammatory stage conducive to rapid development, characterized by fetal expansion and metabolic stability, during which tolerance to microchimerism reaches its zenith. Finally, a second parturition-associated pro-inflammatory and preparatory phase occurs, during which the recruitment of immune cells to the myometrium signals the cessation of functional tolerance and orchestrates the mechanical exigencies of parturition (4, 9).

Interacting with this environment, emerging evidence regarding the microbiota, maternal, placental, and potentially within the amniotic fluid, suggests that the fetus does not develop in a biological vacuum. Microbial stimuli and their derived metabolites are already present and interact with maturing immune cells across diverse anatomical niches (10, 11).

For decades, the “sterile womb” dogma shaped obstetrics, presupposing that the initial microbial encounter occurred exclusively during transit through the birth canal. However, contemporary evidence demonstrates that the fetal immune system is not a passive spectator; while the colonization by viable bacteria remains a subject of methodological debate, there is a consensus that the fetal immune system is actively influenced by the translocation of Pathogen-Associated Molecular Patterns (PAMPs) and metabolites crossing the placental barrier (12). Conversely, studies employing rigorous contamination controls have found no evidence of a resident placental or fetal microbiome in healthy pregnancies, suggesting that microbial signals detected in low-biomass samples may reflect contamination (13, 14). These ligands serve as molecular educators, interacting with pattern recognition receptors (PRRs) within anatomical niches and thereby initiating distinct transcriptional programs that underpin lasting immunological programming even before birth (**Figure 1**).

**Figure 1:**
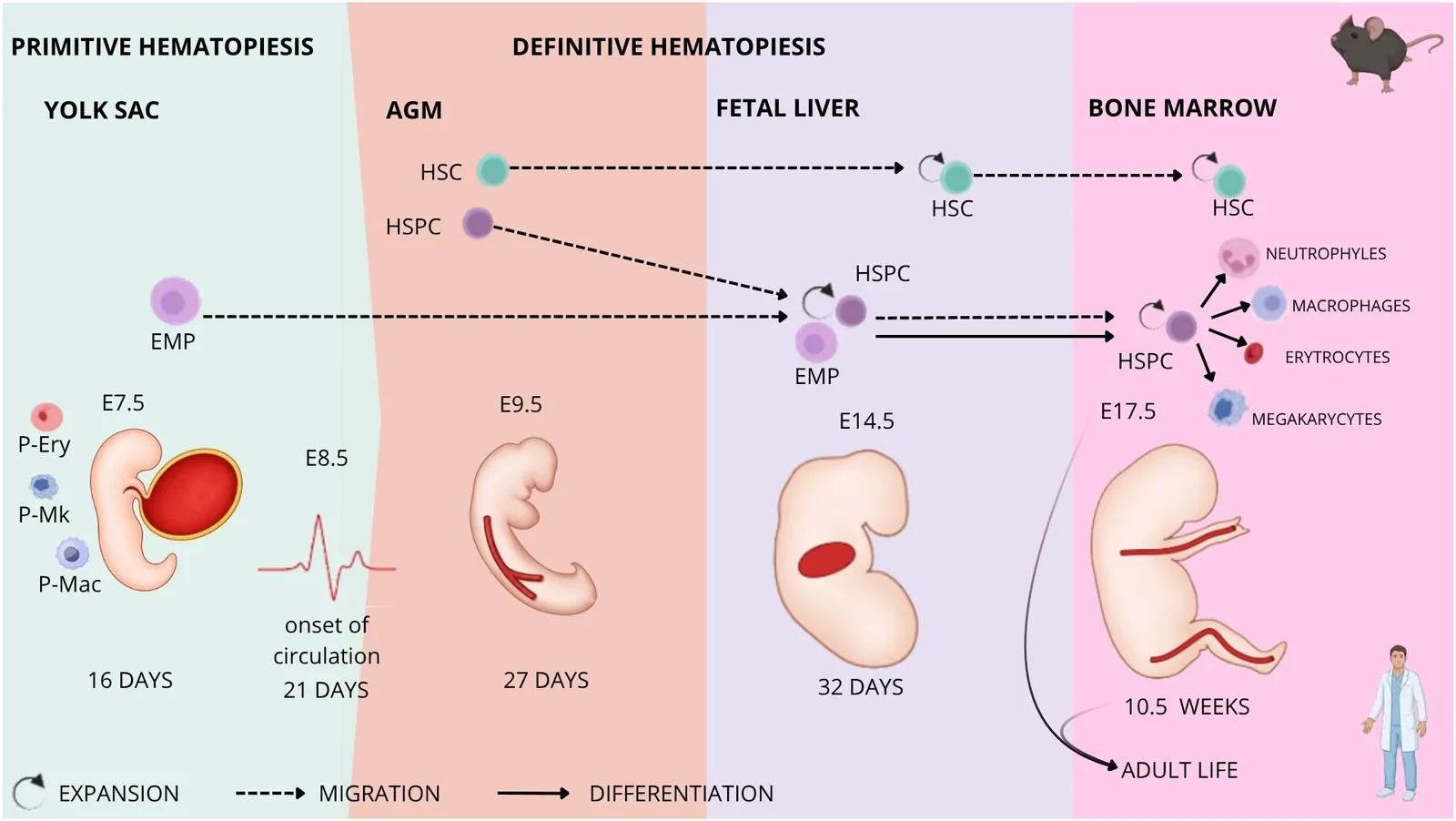
Schematic representation of hematopoiesis during embryonic and fetal development. The diagram depicts the progression from primitive hematopoiesis in the yolk sac to definitive hematopoiesis occurring in the aorta-gonad mesonephros (AGM) region, fetal liver, and eventually the bone marrow. Key developmental stages are highlighted, including the migration and differentiation pathways of hematopoietic stem cells (HSCs), hematopoietic stem and progenitor cells (HSPCs), and erythro-myeloid progenitors (EMPs) toward their respective lineages in adult life.

### 1.2 Hematopoietic ontogeny: niche dynamics and differentiation potential

The hematopoietic system is a complex, integrated network where specialized cells, including erythroid, megakaryocytic, myeloid, and lymphoid lineages, emerge from a common pool of hematopoietic stem and progenitor cells (HSPCs). While adult hematopoiesis is centralized in bone marrow niches, prenatal development follows a distinct spatiotemporal organization (**Figure 2**) (15, 16).

**Figure 2:**
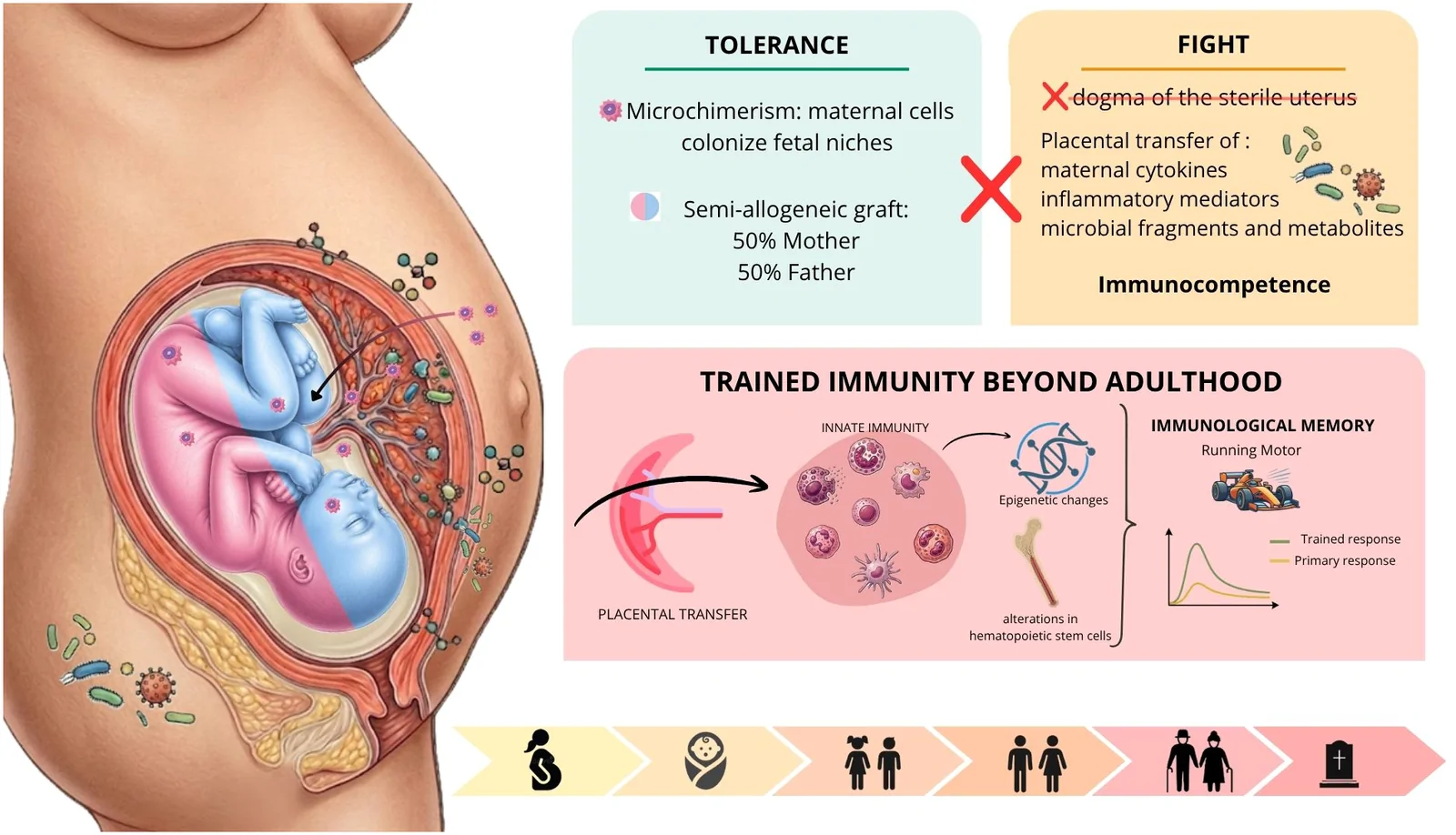
Mechanisms of maternal-fetal immune interaction and developmental programming of trained immunity. The diagram summarizes how maternal microchimerism, modulation of the semi-allogeneic graft (50% mother/50% father), and placental transfer of inflammatory mediators and microbial components influence fetal immune development. This challenges the concept of a completely sterile intrauterine environment and illustrates how these prenatal exposures establish a lasting immunological memory (trained immunity), which can have lifelong health impacts.

During this period, the fetus develops its own hematopoietic machinery through successive migratory waves between distinct anatomical niches, which provide specialized microenvironments that dictate the functional identity and polarization of developing immune cells:

#### 1.2.1 The yolk sac and primitive hematopoiesis

By approximately embryonic day 16, the YS becomes the first site of hematopoiesis, producing a transient population composed predominantly of primitive nucleated erythrocytes, along with smaller numbers of primitive macrophages and megakaryocytes (17, 18).

By the 5th week, a pro-definitive stage emerges as YS mesoderm differentiates into hemogenic endothelium (18). In murine models, this process is critically dependent on SCL/TAL1 and coordinated by a network of hematopoietic transcription factors, including GATA-1, GATA-2, and PU.1 (19, 20). This hemogenic endothelium generates erythro-myeloid progenitors (EMPs), which subsequently give rise to transient myeloid progenitors. KIT ligand (Kitl) signaling is essential for hematopoiesis in both the YS and the Aorta Gonad Mesonephros (AGM), and its disruption results in severe embryonic anemia, compromising the establishment of subsequent fetal liver hematopoiesis (21).

Despite this activity, the YS possesses restricted hematopoietic potential. While early models proposed that YS cells migrate to the liver to establish definitive hematopoiesis, current evidence from pre-circulation specimens reveals that YS-derived CD34+ cells are limited to myeloid and Natural Killer (NK) lineages. In contrast, intraembryonic tissues generate a larger population of CD34+ cells with broad multilineage potential (22, 23).

The third (definitive) stage, occurring between the 7th and 8th weeks of development, is characterized by the emergence of genuine pluripotent hematopoietic stem cells (HSCs) (CD34+, CD144+, CD45+, RUNX1+). These cells originate exclusively from hemogenic endothelium lining the ventral wall of the dorsal aorta through the endothelial-to-hematopoietic transition (EHT) (18, 24, 25).

Given that long-term reconstituting HSCs emerge in the AGM region at day 32 of development, about five days before they are detected in the YS, their subsequent appearance in the YS indicates that these cells originate within the embryo and colonize this extraembryonic tissue via the embryonic circulation. These findings confirm that any HSCs found in the YS have migrated from the embryo. These findings confirm the intraembryonic origin of definitive HSCs and demonstrate that the long-term repopulating activity observed in the YS reflects secondary colonization rather than local HSC generation (23, 26).

#### 1.2.2 The AGM region and hemogenic endothelium

Definitive hematopoiesis originates within the embryo in the AGM region, where HSCs first emerge from the ventral wall of the dorsal aorta beginning at approximately day 32 of development. These cells arise through the EHT, during which emerging hematopoietic cells transiently co-express endothelial and hematopoietic markers, including CD34, VE-cadherin (CD144), CD45, and RUNX1. The EHT is orchestrated by a complex transcriptional network involving NOTCH1, RUNX1, GATA2, and SCL/TAL1, which regulates HSC specification, survival, and maturation. During this process, endothelial cells of the ventral aortic wall give rise to intra-aortic hematopoietic clusters (IAHCs), where nascent HSCs mature before entering the embryonic circulation (22, 24, 27–29).

These IAHCs expand and express key hematopoietic transcription factors, including SCL/TAL1, c-Myb, GATA2, and GATA3 (30). Early studies suggested that the close association between GATA3^+^ and BMP4^+^ mesenchymal cells and emerging IAHCs reflected a possible contribution of the subaortic mesenchyme to definitive HSC development (30, 31). However, subsequent lineage-tracing and live-imaging studies demonstrated that definitive HSCs arise directly from specialized hemogenic endothelial cells through EHT, whereas the surrounding subaortic mesenchyme is now recognized as a specialized hematopoietic niche that provides inductive signals for HSC specification and maturation (25, 32, 33). Additionally, angiotensin-converting enzyme (ACE/CD143) has been identified as an early marker of emerging human HSCs and remains expressed throughout hematopoietic ontogeny (34).

Current evidence localizes this activity to a rare population of CD34^+^CD45^+^c-KIT^+^THY1^+^Endoglin^+^ cells within the ventral wall of the dorsal aorta (28). These cells exhibit remarkable self-renewal capacity, with transplantation assays demonstrating that a single AGM-derived HSC can generate at least 300 daughter HSCs, highlighting the extensive expansion of the definitive hematopoietic compartment during embryonic development (28).

#### 1.2.3 The fetal liver: between expansion and *de novo* generation

Following organogenesis, the fetal liver is initially colonized by primitive hematopoietic cells derived from the YS, which lack the capacity for long-term hematopoietic reconstitution and are therefore considered distinct from definitive HSCs (23, 26).

Subsequently, the fetal liver becomes the principal site of hematopoiesis. Although traditionally viewed as a transient site for the expansion of AGM-derived HSCs, it is now recognized as a highly specialized hematopoietic niche that actively regulates HSC maintenance, self-renewal, lineage commitment, and differentiation (17, 35, 36). This process is supported by fetal liver sinusoidal endothelial cells (FLSECs), which provide essential proliferative signals by secreting hematopoietic growth factors and activating Notch signaling (37). Within this microenvironment, HSCs and multipotent progenitors (MPPs) differentiate into distinct hematopoietic lineages through defined intermediate populations, including a neutrophil-myeloid progenitor characterized by the expression of CD34, SPINK2, MPO, and ELANE (36).

This transition coincides with the decline of hematopoietic activity in the dorsal aorta and a marked expansion of hematopoietic progenitors within the fetal liver, including BFU-E (burst-forming unit-erythroid), CFU-E (colony-forming unit-erythroid), and CFU-GM (granulocyte-macrophage colony-forming unit). The rapid expansion of these lineage-restricted progenitors establishes the fetal liver as the principal hematopoietic organ during prenatal development (17, 22).

During fetal liver hematopoiesis, HSCs and multipotent progenitors (MPPs) undergo progressive transcriptional maturation. Single-cell transcriptomic analyses revealed high HSPA1A expression, consistent with a protective role in maintaining cellular homeostasis during rapid expansion, and relatively low HLA-B expression, reflecting the immunological immaturity of fetal HSCs and defining a molecular profile distinct from that of their adult counterparts (36).

However, despite the presence of phenotypically mature neutrophils, full mobilization of the antimicrobial arsenal, including the extracellular release of granule-derived proteins such as bactericidal/permeability-increasing protein (BPI), remains developmentally restricted during fetal life and early neonatal development. This reflects a state of functional readiness, in which the innate immune system is structurally mature, but its effector functions remain tightly regulated until birth, balancing antimicrobial defense with the need to limit excessive inflammatory responses (38, 39).

As gestation progresses, hematopoietic output in the fetal liver shifts from predominantly erythroid production toward increasingly diverse hematopoietic lineage output, culminating in the definitive transition of hematopoiesis to the bone marrow near birth. In contrast to the greater developmental plasticity of fetal hematopoiesis, the adult bone marrow is characterized by a hierarchical organization in which multipotency is largely confined to the HSC compartment, whereas the progenitor compartment is dominated by unilineage progenitors with limited developmental plasticity (40).

The mechanisms governing hematopoietic lineage commitment remain a subject of intense debate. The classical model proposes a rigid hierarchical organization in which hematopoietic stem cells sequentially generate increasingly lineage-restricted progenitors, with the early segregation of common myeloid (CMP) and common lymphoid progenitors (CLPs) accompanied by the irreversible loss of alternative developmental fates. In contrast, contemporary models support a progressive and continuous process of lineage specification, in which HSCs traverse heterogeneous transitional states, or may differentiate directly into lineage-biased progenitors, including lymphoid-primed multipotent progenitors (LMPPs), under the influence of intrinsic transcriptional programs and extrinsic niche-derived signals (40, 41).

The presence of a VE-cadherin^+^ HSC subset further reinforces the intimate relationship between emerging HSCs and the vascular niche. These cells are enriched in high-proliferative colony-forming cells and long-term hematopoietic activity, whereas their progressive transition to a VE-cadherin^−^ phenotype accompanies hematopoietic maturation while preserving stem cell function. Together, these findings support the concept that the fetal liver is colonized by immature HSCs originating from the AGM region, which subsequently undergo phenotypic maturation within the hepatic microenvironment (26, 35).

#### 1.2.4 Bone marrow colonization

While the long bones remain cartilaginous and lack a defined medullary cavity, they already contain abundant CD68^+^ monocyte-macrophage cells concentrated within the developing diaphysis. Their appearance coincides with active chondrolysis and the invasion of perichondral vascular, osteoblastic, and osteoclastic precursors, initiating the formation of the primary marrow cavity and establishing the microenvironment required for subsequent hematopoietic colonization (42).

The establishment of the marrow niche consolidates with the emergence of primary “logettes,” characterized by arterioles lined with CD34^+^ endothelial cells and α-SMA^+^ smooth muscle cells. Hematopoietic colonization of this nascent cavity initiates around the 10.5th week. Notably, until the 15th week, a predominance of CD15^+^ granulocytes over glycophorin A^+^ erythroblasts is observed. This transient granulocytic predominance likely reflects the active remodeling processes accompanying marrow cavity formation rather than a primary role in host defense (42). The definitive structuring of the medullary cavity in long bones is achieved post-16th week, coinciding with the detection of primitive CD34^+^ progenitors and CFU-GEMM activity. This transition reflects a progressive specialization of hematopoiesis: while the fetal liver remains the hub for multilineage progenitors (myeloid-erythroid-megakaryocytic), the maturing bone marrow shifts toward the dominance of unilineage progenitors (40, 43). Given the limitations in sample acquisition after the 24th week, the precise evolutionary dynamics of this niche until birth remain an area requiring further characterization (44).

#### 1.2.5 Continuous hematopoietic support: the placenta and hemogenic endothelium

Direct analysis of fetal blood confirms that HSPCs circulate throughout gestation with greater proliferative and multilineage potential than their adult counterparts. Between the 12th and 18th weeks of gestation, fetal blood is notably enriched in immature CD34^+^/CD38^−^/Lin^−^ cells that exhibit superior reconstitution capacity in experimental models compared with term cord blood. This kinetic advantage suggests that early fetal HSCs possess either an enhanced intrinsic repopulation program or a higher frequency of primitive stem cells (16, 45, 46).

Since the first successful umbilical cord blood transplantation for Fanconi anemia in 1988, cord blood has become an established source of hematopoietic stem and progenitor cells for transplantation. Its capacity to generate multilineage hematopoietic progenitors and achieve hematopoietic reconstitution provides functional evidence of the substantial repopulating potential of cord blood-derived HSPCs (45–47).

#### 1.2.6 The placenta as a hematopoietic hub and reservoir

The placenta is now recognized as an active hematopoietic niche that harbors CD34^++^CD45^lo^ hematopoietic stem and progenitor cells from early gestation through term. These cells exhibit multilineage differentiation potential *in vitro* and, as early as 6 weeks of gestation, are capable of long-term multilineage engraftment in immunodeficient mice, demonstrating bona fide hematopoietic stem cell activity. Concurrently, hematopoietic activity is also detected in the fetal spleen, although the appearance of CFU-GEMM progenitors occurs later than in the fetal liver and bone marrow, indicating that the spleen plays a comparatively limited role in definitive hematopoietic expansion (48–50).

### 1.3 Ontogeny and functional superiority of fetal hematopoietic stem cells

Beyond cellular migration, the expansion of the prenatal hematopoietic system is also supported by the persistence of hemogenic endothelial potential across multiple fetal organs. Endothelial cells (CD34^+^CD45^−^) isolated from the YS, fetal liver, and fetal bone marrow retain the capacity to generate myeloid and lymphoid cells when cultured under supportive stromal conditions, indicating that hemogenic competence persists well beyond the initial emergence of HSCs in the AGM. This persistent endothelial potential may contribute to the remarkable expansion of the prenatal hematopoietic compartment. Whether adult bone marrow endothelial cells retain this developmental hemogenic program or instead lose it during ontogeny remains unknown (16, 51).

Ontogenetically younger cells are distinguished by their high capacity for expansion and repopulation, along with a specialized sensitivity to growth factors, a recurring pattern in developmental hematopoiesis (52). Marked ontogenic differences in proliferative capacity are observed among hematopoietic stem and progenitor cells. Primitive CD34^+^CD38^−^ cells from umbilical cord blood exhibit higher cloning efficiency, initiate proliferation earlier, and generate approximately sevenfold more progeny than phenotypically equivalent cells from adult bone marrow (53). These findings are consistent with the superior clonogenic competence observed in fetal tissues, including the fetal liver and embryonic bone marrow, compared with umbilical cord blood, adult bone marrow, or mobilized peripheral blood, regardless of surface marker phenotype (54).

This divergence reflects the distinct biological demands of each developmental stage. During embryonic and fetal life, hematopoiesis operates in a highly proliferative state to support rapid somatic growth, expansion of hematopoietic compartments, and increasing oxygen demands. In contrast, adult hematopoiesis relies predominantly on the long-term maintenance of a largely quiescent HSC pool, ensuring lifelong blood production while limiting excessive stem cell turnover. Telomere dynamics indicate that this developmental transition, from a phase of intense stem cell proliferation to one of relative quiescence, is largely established during the first years of postnatal life, when the rate of telomere attrition decreases dramatically, consistent with a marked reduction in HSC turnover (55).

This ontogenic transition is also accompanied by a revision of the classical view of hematopoietic differentiation. Traditionally, hematopoiesis has been depicted as a rigid hierarchical process in which HSCs first bifurcate into CMP and CLP progenitors, then generate progressively lineage-restricted intermediates. However, single-cell transcriptomics, lineage tracing, and fate-mapping studies support a more flexible organization in which HSCs comprise functionally heterogeneous populations and lineage commitment occurs through continuous rather than strictly binary transitions. In this framework, HSCs or their immediate downstream progenitors may directly generate lymphoid-primed multipotent progenitors (LMPPs) or megakaryocyte–erythroid progenitors (MEPs), bypassing some of the classical intermediate stages. Such developmental flexibility may be particularly advantageous during fetal hematopoiesis, where the rapid expansion of blood and immune cell populations is required to support organismal growth (41, 56, 57).

This hematopoietic flexibility is accompanied by precise regulation of neutrophil homeostasis, which depends on a meticulous balance between marrow development, storage, and release into vascular compartments. Unlike the adult, who generates approximately 100 billion neutrophils daily and maintains a marrow storage pool nearly 20-fold larger than the circulating neutrophil pool, the neonate operates with an extremely reduced proliferative pool and high turnover demands (58, 59).

This functional dynamic reflects the biological needs of the fetus, whose hematopoiesis is geared toward accelerated expansion, maintaining up to 75% of circulating CFU-GM in active proliferation. However, this heightened proliferative activity coexists with a markedly reduced marrow neutrophil storage reserve, estimated to be only ~10% of adult values. Following birth, the expansion and maintenance of neutrophil homeostasis become increasingly dependent on microbial colonization, which activates TLR4- and MyD88-dependent signaling pathways that promote IL-17 production by intestinal ILC3 cells, stimulate G-CSF release, and enhance granulopoiesis. This microbiota-driven signaling is therefore essential for establishing the mature neutrophil compartment and effective immune surveillance during early neonatal life (59–63).

At birth, the neonatal immune system undergoes a physiological surge in circulating neutrophils, largely driven by demargination and marrow release induced by stress hormones such as epinephrine, norepinephrine, and cortisol, resulting in the highest leukocyte counts observed during human life (64). However, the persistence of circulating immature neutrophils, which have not yet fully acquired gelatinase granules and secretory vesicles, reinforces that the kinetic superiority of fetal HSCs coexists with qualitative functional immaturity that limits tissue transmigration and antimicrobial responses (65). This dependence on microbiota-derived signals, together with the delicate balance between protection from excessive inflammation and susceptibility to infection, defines the neonatal period as a critical developmental window. Accordingly, interventions that disrupt physiological microbial colonization, such as antimicrobial exposure, impair neutrophil homeostasis, reduce granulopoiesis, and increase susceptibility to neutropenia and sepsis, particularly given the limited neonatal marrow reserve (59–63). Furthermore, this reliance on microbial cues for functional maturation suggests that neonatal-trained immunity may shape long-term immune competence and potentially influence the efficacy of future immunotherapeutic interventions in individuals exposed to early-life dysbiosis.

In summary, the transition from fetal to adult hematopoiesis represents far more than a change in anatomical location; it reflects a fundamental shift in hematopoietic strategy. During fetal life, hematopoiesis operates as an expansion-oriented program that supports rapid cellular proliferation, tissue growth, and immune system establishment. In contrast, adult hematopoiesis is optimized for long-term homeostasis, preserving genomic integrity while maintaining a stable pool of self-renewing hematopoietic stem cells. Understanding this developmental transition and the biological trade-offs associated with the remarkable kinetic capacity of fetal hematopoiesis in the face of postnatal infectious and inflammatory challenges is essential for advancing neonatal medicine, stem cell biology, regenerative therapies, and the rational design of immunomodulatory interventions.

### 1.4 Physical and biological barriers in fetal and neonatal protection: from structural development to microbial colonization

During fetal life, multiple physical and functional barriers are already established. The placenta, fetal membranes, and mucosal surfaces contribute to innate host defense by producing antimicrobial peptides (AMPs), including defensins, secretory leukocyte protease inhibitor (SLPI), elafin, and lactoferrin (66). However, the efficacy of these barriers varies by organ. Preterm infants are particularly vulnerable because of the immaturity of the stratum corneum, delayed establishment of the acidic skin pH, and reduced protection provided by the vernix caseosa, which is rich in antimicrobial peptides and develops predominantly during the final trimester of gestation. Preterm infants are especially vulnerable due to the absence of chemical barriers (acidic pH) and the protective vernix caseosa, which is rich in AMPs and forms only during the final gestational trimester. Although the fetal gastrointestinal epithelium already produces the peptide CRAMP, Paneth cells are functionally immature and begin secreting specific AMPs only postnatally. This developmental gap contributes to neonates’ high susceptibility to pathogens such as Shigella and Salmonella (67). Conversely, respiratory defense relies on ciliary function and the mucociliary apparatus, acting as a physical filter aided by a surface fluid rich in lysozymes and defensins (68).

The microbial barrier, a dynamic layer of biological protection, begins to establish immediately after birth. Neonatal skin is colonized by pioneer commensal microorganisms during a developmental window marked by an influx of regulatory T cells (Tregs), thereby establishing immune tolerance to these newly acquired commensals. Following birth, the intestinal microbiota rapidly expands, although prenatal microbial exposure may already have initiated early host–microbe interactions. The composition of this developing ecosystem is strongly influenced by delivery mode, feeding practices, and antibiotic exposure (69, 70).

The development of this intestinal immune system begins before birth through a genetic program that drives the formation of Peyer’s patches and mesenteric lymph nodes. However, full postnatal maturation depends on the establishment of a commensal microbiota. Resident symbiotic microorganisms provide fundamental signals that not only promote normal immunological development but also influence all subsequent responses. These microbial signals promote the development of isolated lymphoid follicles, support the functional maturation of Peyer’s patches, induce the recruitment of IgA-producing plasma cells and activated lymphocytes to the intestinal mucosa, and shape the differentiation and function of innate lymphoid cells (ILCs) (71–73).

Thus, interaction with the microbiota is not merely passive; it is a fundamental driver of the functional maturation of mucosa-associated lymphoid tissue (MALT) and the developing immune system (74, 75). This concept is supported by observations that cesarean delivery, antibiotic exposure, and infant feeding practices alter early microbial colonization and delay microbiome maturation, changes that are associated with modified immune development (76, 77). Consequently, the developing microbiota acts as an epigenetic instructor: continuous exposure to commensal microorganisms provides metabolic and immunological signals that shape innate immune responsiveness through long-lasting epigenetic remodeling, establishing the basis of trained immunity and enabling more rapid and effective responses to subsequent microbial challenge (76–78). This early-life microbiota–epigenetic axis constitutes a foundational layer of immune programming, influencing baseline immune homeostasis and potentially shaping susceptibility to immune-mediated diseases and responsiveness to future immunomodulatory therapies throughout life.

### 1.5 Ontogeny and specialization of adaptive immunity

Unlike the innate response, adaptive immunity is characterized by specificity for structurally distinct antigens, the requirement for cell differentiation, and the formation of immunological memory. Phylogenetically more recent, it undergoes slow ontogenetic maturation and relies on tight coordination with the innate system. A critical point in this transition is metabolic reprogramming: just as in innate immunity, T lymphocyte activation requires a shift toward aerobic glycolysis to sustain its effector functions (79).

Metabolic reprogramming is now recognized as a shared hallmark of immune activation. In innate immunity, immunometabolic remodeling underlies trained immunity, whereas in adaptive immunity, the metabolic shift toward aerobic glycolysis supports clonal expansion and effector differentiation (78, 79).

αβ T lymphocytes originate from bone marrow-derived progenitors that migrate to the thymus, where they undergo a tightly regulated maturation process. Within the thymic cortex, lymphoid progenitors sequentially differentiate from double-negative (CD4^−^CD8^−^) cells into double-positive (CD4^+^CD8^+^) thymocytes through interactions with thymic stromal cells. Following positive and negative selection, thymocytes downregulate either CD4 or CD8, giving rise to mature single-positive CD4^+^ or CD8^+^ T cells that exit the thymus as naïve lymphocytes. These cells subsequently home to secondary lymphoid organs, where recognition of peptide antigens presented by antigen-presenting cells in the context of major histocompatibility complex (MHC) class II or class I molecules triggers their activation, clonal expansion, and differentiation into specialized effector and memory T-cell subsets (80).

The thymus is the primary site of T-cell development and is most active during fetal and early postnatal life, continuously generating naïve T cells while establishing central immune tolerance. Within the thymic microenvironment, developing thymocytes undergo positive and negative selection, eliminating highly self-reactive clones and generating a T-cell receptor (TCR) repertoire capable of recognizing foreign antigens while remaining tolerant to self-antigens (81). T-cell development begins around the seventh week of gestation with the colonization of the thymus by hematopoietic progenitors that progressively differentiate through distinct developmental stages characterized by the sequential acquisition of T-lineage markers. By approximately the 12th gestational week, mature CD3^+^ T cells are already detectable in the fetal circulation. Nevertheless, the fetal TCR repertoire remains relatively restricted, largely because of reduced terminal deoxynucleotidyl transferase (TdT) activity, which limits N-nucleotide addition during V(D)J recombination and consequently decreases junctional diversity (81–83).

Upon antigen stimulation, naïve CD8^+^ T cells undergo clonal expansion and differentiate into cytotoxic effector cells and long-lived memory cells, thereby playing a central role in host defense against intracellular pathogens (80).

The major CD4^+^ T-cell subsets include Th1, Th17, T follicular helper (TFH), and Treg cells. Th1 cells produce cytokines, such as interferon-γ (IFN-γ), that activate macrophages and promote immunity against intracellular pathogens, whereas Th17 cells secrete IL-17 family cytokines and contribute to host defense against extracellular bacteria and fungi, particularly at mucosal barriers. TFH cells are localized to germinal centers, where they provide essential help to B lymphocytes, promoting affinity maturation, class-switch recombination, and the generation of long-lived plasma cells and memory B cells. In contrast, Treg cells maintain immune homeostasis and peripheral tolerance by limiting excessive immune responses and preventing immunopathology. These cells originate either in the thymus (thymus-derived Tregs; tTregs) or are induced in peripheral tissues following antigen exposure (peripherally derived Tregs; pTregs) (84–87).

Mature circulating T lymphocytes become detectable by approximately 15–16 weeks of gestation, after the establishment of the major innate lymphocyte populations. Thereafter, the TCR repertoire undergoes progressive diversification throughout fetal development, accompanied by the expansion of thymus-derived regulatory T (tTreg) cells, reflecting the parallel maturation of adaptive immunity and mechanisms of immune tolerance (88, 89).

The fetal adaptive immune system is intrinsically biased toward the generation of Treg cells, a process promoted by exposure to maternally derived cells and non-inherited maternal antigens (NIMAs) that cross the placenta during pregnancy. This tolerogenic program is essential for maintaining maternal-fetal tolerance, supporting maternal microchimerism, and establishing long-lasting tolerance to both maternal alloantigens and fetal self-antigens. By suppressing potentially harmful immune responses during development, fetal Tregs prevent immune-mediated damage to developing tissues while preserving immune homeostasis. Collectively, these findings indicate that the fetal hematopoietic environment is developmentally programmed to favor immunological tolerance over effector immunity (90–92).

Clinically, newborns exhibit a reduced propensity to mount Th1 responses, reflecting both the limited production of Th1-polarizing cytokines, particularly IL-12, by neonatal antigen-presenting cells and the intrinsically reduced capacity of neonatal T cells to produce IFN-γ (84, 93). Consequently, immunity against intracellular pathogens is generally less effective during early life. However, this limitation is not absolute, as strong inflammatory stimuli, including BCG vaccination and congenital Cytomegalovirus (CMV) infection, can induce robust Th1-type differentiation, although fetal T cells often retain functional constraints compared with adult responses (94). Furthermore, the neonatal cytokine milieu supports the differentiation of Th17 and TFH cells under appropriate stimulatory conditions (84). Studies have shown that a subset of fetal αβ T lymphocytes acquires memory-like phenotypes *in utero* and can produce Th1-, Th2-, or Th17-associated cytokines, although the stimuli driving this differentiation, including the possible contribution of microbial antigens, remain incompletely understood (95).

Mature B lymphocytes can be detected in the fetal liver from approximately the 8th week of gestation. Between 12 and 15 weeks of gestation, B-cell development shifts to the bone marrow, which becomes the primary site of B lymphopoiesis. After leaving the bone marrow, immature/transitional B cells migrate through the bloodstream to secondary lymphoid organs, where they complete maturation into naïve B cells and subsequently become activated upon antigen encounter (96).

The primary function of B lymphocytes is to mediate humoral immunity through antibody production. Following antigen recognition, antigen-specific B cells internalize and process protein antigens, presenting peptide–MHC class II complexes to CD4^+^ T lymphocytes. This interaction promotes germinal center formation and drives B-cell proliferation and differentiation. Initially, activated B cells produce IgM, establishing the primary humoral response. Subsequently, signals delivered by T follicular helper (Tfh) cells induce immunoglobulin class-switch recombination, somatic hypermutation, and affinity maturation, enabling the generation of high-affinity antibodies such as IgG, IgA, and IgE. Following the germinal center reaction, B cells differentiate into short-lived plasma cells, which rapidly secrete antibodies; long-lived plasma cells, which sustain antibody production primarily within bone marrow survival niches; and memory B cells, which mediate faster and more robust antibody responses upon subsequent exposure to the same antigen (97).

During fetal life, somatic hypermutation and immunoglobulin class-switch recombination already occur, contributing to the early diversification of the B-cell receptor repertoire before birth (89). Although neonatal antibody responses vary with the nature of the antigen and the immunological stimulus, vaccination at birth can induce robust humoral immunity and long-lasting immunological memory, thereby supporting enhanced responses following booster immunization in early infancy (98).

Although the intrauterine environment is relatively protected, maternal proteins, antibodies, cells, and other foreign antigens can cross the placental barrier during fetal development (99, 100). Despite the age-dependent maturation of T- and B-lymphocyte compartments, characterized by a predominance of naïve lymphocytes, limited memory populations (101) and a predominantly tolerogenic immune profile (90), the fetus retains the capacity to mount functional immune responses against infectious challenges, although these responses differ qualitatively from those of adults (39) This developmental program reflects a finely regulated balance between immune tolerance and protective immunity, which must rapidly adapt after birth as the newborn encounters a broad array of environmental antigens.

The mechanisms governing the maturation of effector B-cell responses remain incompletely understood but are thought to involve reduced expression of costimulatory molecules and complement receptors on neonatal B cells, together with less efficient interactions between B cells and T follicular helper (TFH) cells, resulting in suboptimal germinal center reactions. These developmental constraints likely contribute to the age-dependent efficacy of humoral responses to different vaccines. Whereas immune responses to some vaccines, such as poliovirus vaccines, progressively improve with age, hepatitis B vaccination administered at birth can elicit robust antibody responses and durable immunological memory, despite qualitative differences from adult immune response (89, 98, 102).

The production of high-affinity antibodies by newborns is limited in the first weeks of life because germinal center reactions and affinity maturation remain functionally immature, making the transplacental transfer of maternal antibodies essential during gestation. IgG is the primary isotype transported across the placenta by the neonatal Fc receptor (FcRn), thereby providing passive protection against pathogens to which the mother has been previously exposed through natural infection or vaccination. This mechanism provides the biological basis for maternal immunization during pregnancy, an effective strategy for protecting newborns against infections such as tetanus and pertussis (97, 103, 104).

Following birth, maternally acquired IgG levels decline progressively, particularly in preterm infants, in whom transplacental antibody transfer is reduced, increasing susceptibility to bacterial infections. Although maternal antibodies may attenuate the magnitude of vaccine-induced humoral responses during the first months of life, they do not impair the establishment of B- and T-cell immunological memory. Consequently, immunological priming occurs even in the presence of maternal antibodies, allowing more robust responses following booster immunizations (88, 105, 106). Furthermore, accumulating evidence indicates that the maternal microbiome helps shape the initial immunological landscape of the fetus and newborn through microbial metabolites, immune mediators, and maternal-derived signals, thereby influencing immune maturation and the establishment of long-term immune homeostasis (107–110).

### 1.6 Fetal and neonatal immunological development: from tolerance to active response

During pregnancy, the fetal immune system develops within a relatively protected and antigen-limited environment. At the same time, pregnancy represents an immunological paradox because the fetus is semi-allogeneic, expressing paternally derived antigens that are foreign to the maternal immune system (111). Consequently, fetal immune development must reconcile the acquisition of self-tolerance with the establishment of tolerance to maternally derived and selected paternal alloantigens without compromising the capacity to generate protective immune responses. This developmental program results in a predominantly tolerogenic immune profile during fetal life (111, 112).

Treg cells expressing the Foxp3 transcription factor constitute a specialized subpopulation of CD4+ T lymphocytes and play an essential role in maintaining immune tolerance (113). Foxp3 expression is the defining molecular feature of these cells and is indispensable for the acquisition and maintenance of their regulatory phenotype. Whereas conventional T cells mediate effector immune responses, Foxp3+ Treg cells suppress immune activation, limiting excessive inflammation and preserving immune homeostasis. Unlike conventional T cells, thymic Treg cells are selected following relatively high-affinity recognition of self-antigens, a developmental process that endows them with dominant suppressive capacity and enables the maintenance of self-tolerance while preventing autoimmune responses (114).

Thymic Treg-cell development occurs through a two-step process. Initially, TCR signaling together with costimulatory signals promotes the generation of CD25^+^Foxp3^−^ Treg precursors. Subsequently, IL-2 signaling induces Foxp3 expression, driving the differentiation of these precursors into mature thymic (natural) Treg cells (115, 116).

From approximately the 14th week of gestation, differentiated T cells leave the thymus and migrate to peripheral lymphoid organs (117). Studies have shown that both fetal circulation and lymphoid tissues exhibit significantly higher proportions of Treg cells than in newborns and adults (90, 118). Evidence further suggests that fetal T cells arise from a developmentally distinct lineage from that present after birth, exist only for a limited period during intrauterine life, and are programmed to respond in a tolerogenic manner (91).

Collectively, these findings indicate that the development of the fetal immune system is primarily driven by the need to establish tolerance to the organism itself and, possibly, to maternal antigens. In contrast, after birth, the immune system becomes shaped by the need to recognize and respond to potentially harmful foreign antigens (91, 112).

Recent studies demonstrated that the frequency of fetal Treg cells is strongly and specifically correlated with maternal Treg frequency, a relationship not observed for Th1, Th2, or Th17 cells. This association cannot be explained by shared genetics, as no comparable correlation was found with paternal Treg frequencies, nor by maternal cell transfer, since maternal Treg cells were absent from umbilical cord blood. These findings provide compelling evidence that maternal and fetal Treg compartments are developmentally coordinated through mechanisms that remain incompletely understood (119).

Although these observations were obtained from term pregnancies, it remains unclear whether this maternal–fetal Treg synchrony is established earlier during gestation. Evidence indicates that preterm newborns (24–31 weeks of gestation) exhibit higher frequencies of Treg cells in cord blood than term newborns (120). These findings are consistent with previous studies demonstrating an enrichment of Treg cells in fetal circulation and lymphoid tissues between the 12th and 20th weeks of gestation (90, 118). Nevertheless, the physiological dynamics governing maternal and fetal Treg-cell populations during early pregnancy remain poorly understood, largely because most available studies have focused on pathological conditions, including spontaneous preterm labor and other pregnancy complications (121, 122).

The mechanisms responsible for the alignment between maternal and fetal Treg cell compartments are not yet fully understood. One hypothesis suggests that the maternal immune system, shaped by environmental exposures, may act in ‘calibrating’ the fetal immune system, favoring its adaptation after birth. Alternatively, the fetal immune system, driven by the establishment of tolerance, could influence maternal immune regulation to ensure a successful pregnancy (119). Additionally, fetal-maternal microchimerism, characterized by bidirectional cell exchange between mother and fetus, may contribute to this phenomenon (123, 124).

Cytokines are important mediators of immune communication during gestation. Pregnancy is accompanied by dynamic changes in systemic cytokine profiles, involving both immunoregulatory cytokines, such as IL-10 and TGF-β, and pro-inflammatory mediators, including IL-6 and IFN-γ, that vary with gestation (125–127). Among these molecules, IL-10 is considered particularly relevant for maintaining pregnancy (128). Although the ability of various cytokines to cross the placenta remains controversial (129–131), a significant correlation was observed between mother and fetus in serum IL-10 levels and in IL-10 production by CD4+ and CD8+ T lymphocytes, suggesting coordinated regulation of IL-10 production in maternal and fetal immune compartments (119). Furthermore, IL-2 is indispensable for Treg cell survival and expansion, whereas TGF-β primarily contributes to the induction and maintenance of peripheral Treg cells. Conversely, inflammatory cytokines, particularly IL-6, antagonize Treg differentiation and favor effector T-cell responses, whereas the effects of TNF-α on Treg biology appear to be context dependent (132–135). Although the role of IL-10 in Treg biology is not yet fully elucidated, several studies point to its importance in maintaining the homeostasis and suppressive activity of these cells (119, 136, 137).

Pregnancy hormones also play important immunomodulatory roles in regulating Treg cells. Human chorionic gonadotropin (hCG), produced by syncytiotrophoblasts, can attract Treg cells to the decidua and modulate their function (138, 139). Similarly, estradiol (E2) and estriol (E3), the main pregnancy estrogens, promote Treg cell proliferation and activity, with E3 exhibiting potent immunomodulatory properties (140–144). Progesterone also contributes to immune tolerance by inhibiting Th1 responses and reducing effector CD4+ T cell activity, and by stimulating Treg cell induction (145–147). Notably, hCG, E2, E3, and progesterone are also associated with increased IL-10 production (148–150). The strong correlation between maternal and fetal hormonal concentrations suggests that pregnancy-associated hormones may contribute to the coordinated regulation of maternal and fetal immune responses. Given their well-established effects on Treg differentiation and IL-10 production, these hormones have been proposed as potential mediators of maternal–fetal immune communication, although direct evidence linking them to the coordinated regulation of maternal and fetal Treg-cell compartments remains lacking (119, 151, 152).

Recent evidence also indicates that maternal environmental signals, especially those related to microbiota, can influence fetal immune development. Experimental animal studies have provided evidence that maternal bacteria or bacterial components can reach the fetus under specific conditions, challenging the traditional concept of a completely sterile intrauterine environment (12, 153) In humans, the detection of microorganisms in meconium, cord blood, and fetal membranes supports the possibility of microbial transmission during intrauterine life (154, 155). Beyond direct microorganism transfer, metabolites produced by the maternal microbiota, such as short-chain fatty acids (SCFAs), may contribute to the induction and maintenance of Treg cells (156–158). Therefore, maternal microbial exposure appears to represent yet another important mechanism for fetal immune programming and for modulating immune tolerance during gestation.

### 1.7 The ontogeny of neonatal innate immunity

Innate immunity represents the most ancestral form of defense in multicellular organisms and is essential for immediate host protection (159). Characterized by a rapid, nonspecific response and comprising a complex network of cells and soluble molecules, it uses PRRs to detect microbial signatures (PAMPs) and trigger cascades of cellular reactions that lead to the production of effector molecules. Recently, our understanding of this system has expanded through its interface with cellular metabolism and nutritional immunity: activation of PRR-dependent PI3K/Akt/mTOR signaling promotes a metabolic shift toward aerobic glycolysis, a process that not only sustains phagocytic function but also supports the metabolic and epigenetic reprogramming that underlies “trained immunity”. This involves epigenetic modifications that lead to long-term alterations in innate immunological memory. Beyond its direct antimicrobial functions, innate immunity provides the essential bridge between innate and adaptive immune responses, primarily through antigen presentation and the expression of co-stimulatory molecules, thereby guiding the maturation of a specialized immune response (160, 161).

Neonatal innate immunity should not be regarded as an immature or weakened version of the adult immune system, but rather as a developmentally specialized program optimized for the transition from the relatively protected intrauterine environment to the microbe-rich extrauterine world.

#### 1.7.1 Soluble components: the complement system

The complement system consists of a cascade of enzymatic proteins that deposit various components onto the surface of microbes, initiating opsonization, phagocytosis, or the direct lysis of pathogens. This system illustrates the functional transition from fetus to adult: the activity of the classical (CH50) and alternative (AP50) pathways is significantly reduced in the newborn, reaching approximately 50% to 70% of adult levels, and the terminal components of the membrane attack complex (MAC), C8 and C9, show a particularly sharp dependence on gestational age, with the lowest levels observed in the most premature infants. It is noteworthy that the complement proteins that show a great, age-dependent change in plasma concentration (most of them) are produced in the liver. In contrast, C7, which is largely neutrophil-derived rather than synthesized in the liver, is only modestly reduced in preterm infants, suggesting that the reduced activity of the classical and alternative pathways is due to hepatic immaturity (162–164).

In parallel, antimicrobial proteins and peptides (APPs) constitute key soluble effectors of fetal and neonatal innate immunity. Several APPs exhibit gestational age-dependent expression, including lactoferrin, bactericidal/permeability-increasing protein (BPI), cathelicidins, and defensins. In preterm and term newborns, reduced concentrations of these molecules, together with diminished complement activity and impaired neutrophil extracellular trap (NET) formation, contribute to increased susceptibility to bacterial and fungal infections in early life (163, 165, 166).

#### 1.7.2 Cellular effectors

Cells that comprise the innate immune system primarily include myeloid lineages—such as granulocytes, monocytes, macrophages, and dendritic cells (DCs)—as well as distinct populations of innate lymphocytes, including NK cells, ILCs, invariant natural killer T (iNKT) cells, and γδ T lymphocytes (39). Evidence suggests that, during specific developmental stages, fetal hematopoietic stem and progenitor cells generate immune cells with functional properties distinct from those of their adult counterparts, supporting the concept that fetal immunity reflects a developmentally specialized program rather than simple immunological immaturity (167).

Neutrophils constitute the first line of cellular defense against invading microorganisms. Their ontogeny begins in the fetal liver as early as the fifth week of gestation, followed by a progressive shift of granulopoiesis to the bone marrow from mid-gestation onward. During fetal life, circulating neutrophils are relatively scarce, whereas neutrophil progenitors with robust colony-forming capacity are abundant. This developmental profile changes near birth, coinciding with a marked increase in granulocyte colony-stimulating factor (G-CSF), which promotes granulocytic differentiation and contributes to the rapid expansion of the circulating neutrophil pool (76).

From a functional perspective, these cells exhibit several deficiencies relative to their adult counterparts, including low levels of lactoferrin and BPI, as well as impaired chemotaxis, respiratory burst activity, and NET formation. In cases of extreme prematurity, these functional limitations are further exacerbated by reduced expression of essential signaling molecules, including TLR2, TLR4, and MyD88. Moreover, the rapid exhaustion of these cells during infectious processes helps explain the recurrent neutropenia observed in neonates (76, 168).

Like neutrophils, monocyte generation and their release into the fetal circulation begin following the colonization of the fetal liver by self-renewing hematopoietic stem cells (88). Although multiple monocyte populations have been described, three major subsets are recognized in humans based on phenotypic and functional characteristics: classical (CD14++CD16^-^), intermediate (CD14^++^CD16^+^), and non-classical (CD^14dim^CD16^+^) monocytes (169). Classical monocytes exhibit a predominantly pro-inflammatory profile and are rapidly recruited to sites of infection, although this recruitment is less efficient in neonates (76, 88). In contrast, non-classical monocytes are more closely associated with anti-inflammatory and tissue repair functions, whereas intermediate monocytes display enhanced inflammatory responses, particularly through TLR4 signaling (170).

Age-related functional differences are also observed among these subsets. Umbilical cord blood monocytes exhibit reduced expression of TLR4 and MyD88, and decreased NF-κB activity, resulting in reduced production of pro-inflammatory mediators following TLR stimulation. However, IL-6 and IL-10 production may remain comparable to or even exceed that observed in adults, suggesting a qualitatively distinct functional profile (171).

Furthermore, fetal monocytes exhibit increased STAT protein phosphorylation in response to IFNγ, predominantly promoting the expression of genes associated with antimicrobial defense rather than antigen presentation or inflammation. Neonatal macrophages also display reduced responsiveness to IFNγ and diminished phagocytic capacity against Escherichia coli, particularly in preterm newborns. Collectively, these alterations may contribute to the increased susceptibility of neonates to bacterial infections (76, 171).

DCs are professional antigen-presenting cells that develop from dedicated bone marrow-derived precursors and occupy a central position at the interface between innate and adaptive immunity. These cells are specialized in antigen processing and presentation and are essential for the development of immunological memory and tolerance (76, 172). DCs are primarily classified into conventional dendritic cells (cDCs) and plasmacytoid dendritic cells (pDCs). Under inflammatory conditions, they may also arise from monocytes, giving rise to monocyte-derived dendritic cells (MoDCs), which share functional properties with other DC subsets despite their distinct origin (172).

The developmental regulation of DC subsets and their functional specialization across different stages of life remains incompletely understood. In mice, cDC1 cells predominate during early life, whereas cDC2 cells become increasingly prevalent in adulthood. Neonatal DCs also exhibit lower expression of MHC class II and co-stimulatory molecules, resulting in reduced antigen-presenting capacity and diminished T-cell priming compared with adult DCs (76, 171, 172).

Furthermore, the responses of human cDCs to TLR stimulation vary according to age. In newborns, these cells produce lower levels of IL-12p70, a cytokine essential for Th1 polarization, while producing higher levels of IL-23, thereby favoring Th17-associated responses. This functional profile persists until adolescence and has been linked to epigenetic regulation of IL12A (IL-12p35) transcription, illustrating how developmental epigenetic programming shapes the balance between Th1 and Th17 immunity during immune maturation (171). Immune ontogeny is governed not only by cellular maturation but also by stable epigenetic mechanisms that influence immune responsiveness throughout life. Beyond these age-dependent differences in cytokine production, human fetal DCs also exhibit a distinct immunoregulatory profile. McGovern et al. demonstrated that fetal cDCs retain the capacity to respond to innate immune stimuli while promoting Treg induction and suppressing T-cell TNF-α production through arginase-2-mediated depletion of L-arginine. This mechanism may contribute to maintaining prenatal immune tolerance while preserving fetal DCs’ capacity to respond to microbial stimuli (173).

PDCs from umbilical cord blood also exhibit reduced responses to TLR7 and TLR9 stimulation, with lower production of IFNα and IFNβ in response to viruses such as HCMV, HSV-1, and RSV. This phenomenon appears to be associated with reduced nuclear translocation of the transcription factor IRF7. Similar developmental mechanisms have also been described in murine models, although the extent to which these age-dependent differences influence susceptibility to infection and vaccine responses in humans remains incompletely understood (171, 172).

Innate lymphocytes are lymphoid cells distinct from conventional T and B lymphocytes of the adaptive immune system. They respond rapidly to infection without requiring extensive clonal expansion and contribute to early host defense by recognizing conserved molecular or stress-associated signals. Some innate lymphocytes express rearranged T- or B-cell receptors, whereas others, known as ILCs, lack these receptors. ILCs comprise cytotoxic NK cells as well as the ILC1, ILC2, and ILC3 subsets (174, 175). Importantly, the degree of “innateness” is closely linked to ontogeny. Rather than representing a homogeneous population, innate lymphocyte subsets emerge at distinct developmental stages, with several populations being preferentially generated during fetal life while others continue to develop after birth, reflecting specialized functions during immune maturation (174).

NK cells exert potent cytotoxic activity and rapidly produce inflammatory cytokines, including IFN-γ and TNF-α, following stimulation by myeloid-derived cytokines or activating receptors. NK cells first emerge during fetal liver hematopoiesis and are found in numbers equal to or greater than those observed in adults in newborns. Despite their elevated cytokine production, neonatal NK cells exhibit reduced cytotoxic capacity against certain viruses (76, 88, 176, 177). This functional maturation is accompanied by a developmental shift from immature CD2^−^ NK cells, which predominate in cord blood and fetal tissues, toward CD2^+^ NK cells with enhanced effector functions, including Fas ligand-mediated cytotoxicity (176).

All major ILC subsets have been identified in the fetal liver. Among innate lymphocytes expressing rearranged receptors, invariant NKT (iNKT), MAIT, and γδ T cells are particularly notable. iNKT cells are abundant in the fetal thymus and can produce TNFα and IFNγ at birth. MAIT cells, which are present in fetal mucosal tissues, recognize microbial riboflavin-derived metabolites presented by MR1, suggesting an early role in antibacterial defense. Similarly, γδ T cells are generated in successive developmental waves during fetal life, acquiring tissue-specific functional programs before birth and contributing to early immune surveillance at epithelial barriers (88, 174).

γδ T cells arise early during fetal development and constitute one of the earliest T-lymphocyte populations to acquire effector function. During the second trimester, Vγ9Vδ2 cells predominate in the fetal circulation and recognize phosphoantigens derived from the microbial methylerythritol phosphate (MEP) pathway, enabling rapid responses to conserved microbial metabolites independently of classical MHC presentation (178, 179). As gestation progresses, the composition of the γδ T-cell compartment changes, with Vδ1-expressing cells becoming increasingly predominant toward birth. Consistent with their role in early host defense, robust TNF-α- and IFN-γ-producing γδ T-cell responses have been described in newborns with congenital cytomegalovirus infection, highlighting their contribution to neonatal antimicrobial immunity (174, 178–180).

Among innate-like lymphocytes expressing rearranged B-cell receptors are marginal zone (MZ) B cells, which develop during fetal life and undergo early diversification of the IgM repertoire. However, young children exhibit a limited capacity to produce antibodies against polysaccharide antigens, contributing to their increased susceptibility to encapsulated bacteria such as Haemophilus influenzae type b, Streptococcus pneumoniae, and Neisseria meningitidis. This finding indicates that, despite their early ontogeny, MZ B cells do not achieve full functional maturity during the first years of life (88, 181, 182).

The early development of innate lymphocyte populations during fetal life demonstrates that newborns are equipped with specialized effector mechanisms capable of responding rapidly to both pathogenic and commensal microorganisms immediately after birth. In addition to their immediate protective functions, accumulating evidence indicates that certain innate immune cell populations, including monocytes, macrophages, NK cells, and hematopoietic progenitors, can undergo long-lasting functional reprogramming following microbial or inflammatory stimulation, a phenomenon termed trained immunity. Whether similar mechanisms operate broadly across all innate lymphocyte subsets during fetal and neonatal life remains incompletely understood (183, 184).

#### 1.7.3 Trained immunity

Over millions of years of evolution, the ability to store information about a prior infectious insult to optimize survival has shaped the defense systems of living organisms. Although classical immunological memory, based on the genetic rearrangement of T and B lymphocytes, is exclusive to vertebrates, which represent at most 3% of the planet’s species, the remaining 97% of multicellular organisms rely strictly on innate immunity. Far from being rigid and unadaptable systems, invertebrates and plants have been shown to develop immunological memory in their defenses following priming by a prior pathogenic exposure, ensuring protection against heterologous reinfections (185–190). It was discovered that this ancient, antigen-agnostic mechanism has been highly conserved throughout evolution and remains active in vertebrates. In these animals, innate immune cells retain this property through robust metabolic and epigenetic reprogramming, a phenomenon now well-established in the literature as innate immune memory or trained immunity (160, 191).

A precise understanding of host defense adaptations requires a clear distinction among three functional processes: differentiation, priming, and trained immunity. Differentiation is a one-way street: the cell is definitively transformed, altering its morphology and assuming a fixed function. Priming, on the other hand, serves as an immediate, short-term alert; the primary stimulus remains active within the organism when the second encounter occurs, generating a cumulative or synergistic effect. In contrast, trained immunity stands out as a flexible and reversible state: once the pathogen or antigen is cleared, the cell returns to its basal resting state. The key difference is that, despite returning to normal, it undergoes an internal reprogramming that leaves a sort of “armed trigger” or “engine running,” allowing a future attack, even if caused by a completely different agent, to unleash a much faster and more powerful immune response (184).

Trained immunity represents a watershed moment in immunology by consolidating the concept of memory within the innate immune system. Unlike classical adaptive memory, which relies on clonal expansion at the single-cell level, training occurs at the cell-population level through reprogramming of hematopoietic stem and progenitor cells in the bone marrow and of mature immune cells distributed throughout the body. Furthermore, it is completely antigen-agnostic, conferring broad-spectrum heterologous protection against infections distinct from that of the initial stimulus (78, 184, 192).

The induction of this functional state is triggered by the recognition of microbial patterns by cellular receptors, which activate intracellular signaling pathways and transcription factors related to the inflammatory response. Consequently, a broad metabolic and epigenetic reorganization occurs, characterized by increased chromatin accessibility via histone modifications and persistent transcriptional remodeling. A portion of these alterations persists even after the removal of the initial stimulus, enabling faster and more intense immune responses upon subsequent exposures (193–195).

Recent studies demonstrate that Krebs cycle metabolites play a central role in this process by acting as cofactors for epigenetic enzymes. Stimuli that induce trained immunity, such as β-glucan and BCG, increase aerobic glycolysis via the Akt-HIF-1α-mTOR pathway and redirect cellular metabolism toward the accumulation of metabolites such as fumarate, succinate, and mevalonate, which can sustain long-lasting epigenetic changes. This interaction between metabolism and epigenetics maintains genes involved in immune defense in a state of enhanced transcriptional readiness, favoring a heightened response to new immunological challenges (78, 194, 196, 197).

Although the vertebrate immune system exhibits a high capacity for adaptation throughout life, evidence indicates that certain immunological parameters are established during critical developmental windows. During these periods, environmental stimuli can induce long-lasting adjustments in immune regulation, favoring specific responses over others. Recent studies have demonstrated that inflammatory signals can persistently remodel hematopoietic stem cells and other immune components, extending the concept of trained immunity beyond adulthood. In this context, pregnancy represents a period of heightened biological plasticity, during which maternal factors may influence the development of the offspring’s immune system through metabolic, epigenetic, and cellular mechanisms. Despite growing epidemiological support for this concept, the mechanisms underlying immune programming and the specific immune components susceptible to this process remain poorly understood (198). Elucidating these pathways is essential for identifying novel immunogenomic biomarkers, which may ultimately predict how early-life environmental exposures influence the efficacy of precision immunotherapeutic interventions in adulthood.

### 1.8 Immunological programming

A substantial body of epidemiological evidence indicates that environmental exposures during critical developmental periods exert long-lasting effects on the immune system. Reduced exposure to microorganisms early in life has been associated with an increased incidence of allergies and autoimmune diseases, whereas early exposure to environments rich in microbial stimuli appears to exert a protective effect (199–202). Furthermore, infections, alterations in the microbiota, and antibiotic use during the first years of life have been linked to changes in the risk of developing inflammatory diseases later in life (203–206). However, this relationship is not universal, as certain early-life viral infections have been associated with an increased susceptibility to future inflammatory disorders, including asthma and multiple sclerosis (207, 208). Collectively, these findings support the concept of immune programming, whereby early-life experiences can durably shape immune function.

Immune programming is often manifested through alterations in the response bias of specific cell types upon stimulation, via the process of polarization, which begins long before birth and can be maintained over the long term. Experimental studies have demonstrated that stimuli present during pregnancy can directly influence the polarization of immune cells. This phenomenon occurs because maternal immune activation (MIA) induces a lymphoid bias in fetal hematopoietic stem and progenitor cells, influencing postnatal immune output by promoting the expansion and hyperactivation of innate lymphocytes (209). Moreover, MIA has recently been shown to induce long-term alterations in microglia, characterized by changes in immune reactivity and transcriptional profiles that affect neural circuits and their functions in mice (210). Extending these findings, high-resolution spatial transcriptomic analyses have shown that both maternal immune activation and maternal microbiome depletion reshape the neuroimmune landscape of the developing brain, producing sex-specific alterations in immune signaling networks. Notably, both perturbations affected the CXCL12/CXCR7 chemokine network, suggesting a potential shared mechanism linking maternal gut–immune disturbances to abnormalities in neural progenitor development (211). Beyond these transcriptional changes, recent evidence indicates that MIA also reshapes the epitranscriptomic landscape of the developing brain. Alterations in m6A RNA modifications and in key components of the m6A regulatory machinery have been identified following MIA, including increased expression of the m6A demethylase FTO in specific neural cell populations. These findings identify RNA modifications as an additional regulatory layer through which maternal inflammation may influence neurodevelopment and long-term developmental programming (212).

Evidence of immune training in the absence of vertical transmission is not restricted to experimental models. Human studies indicate that chronic maternal infections, such as those caused by HIV-1 or hepatitis B virus, can alter the fetal immune environment through increased circulating cytokine levels, thereby modulating the development and responsiveness of the offspring’s immune system (213). Among these mediators, IL-17 has been implicated not only in immune programming but also in alterations of fetal neurodevelopment (214). Supporting this concept, infants exposed *in utero* but uninfected with HIV, malaria, or hepatitis B virus exhibit persistent alterations in innate immune responses, characterized by modified cytokine production profiles upon Toll-like receptor (TLR) stimulation (215–219).

Remarkably, this phenomenon may occur even in the absence of vertical pathogen transmission and, in some cases, even without active maternal infection, as prior maternal BCG immunization has also been associated with alterations in the innate immunity of the offspring (220) Together, these findings indicate that the fetal immune system is capable of responding indirectly to maternal immune stimuli, establishing long-lasting adaptations that may influence postnatal immunity.

Although the mechanisms involved in this process remain incompletely understood, it is believed that the transfer of maternal cytokines and other inflammatory mediators across the placenta may modulate fetal immune development. Investigation of this phenomenon in humans remains limited due to the ethical and methodological challenges inherent to studying the maternal–fetal interface during pregnancy. Ex vivo experiments using human placentas suggest that placental cytokine transfer is reduced during the later stages of gestation (129, 130, 221–224).

Conversely, experimental models suggest that certain maternal cytokines can cross the placenta, particularly during early gestation, thereby influencing the development of immune responses in the offspring. Similarly, pathogen-associated Toll-like receptor (TLR) ligands also appear to reach the fetal compartment during this period. However, information remains scarce regarding which maternal mediators participate in this process and the mechanisms by which they modulate fetal immunity (224).

In addition to exposure to maternal inflammatory mediators, alterations at the maternal–fetal interface resulting from infections during pregnancy may also modulate fetal immunity. Experimental and clinical evidence demonstrates that placental inflammatory processes, such as chorioamnionitis and placental malaria, can induce cytokine production and alter the differentiation and activation of fetal immune cells, even in the absence of detectable fetal infection. These findings suggest that maternal inflammation can trigger fetal immune responses through indirect mechanisms, thereby contributing to immune programming before birth (225, 226).

Studies have also demonstrated that embryos possess intrinsic defense mechanisms, including the constitutive expression of interferon-stimulated genes in embryonic stem cells, which confer protection against viral infections and potentially deleterious endogenous genomic effects during development (227, 228). Beyond these intrinsic mechanisms, evidence indicates that the fetal immune system contains innate immune components capable of recognizing and responding to microorganisms, despite the fetus’s high susceptibility to infections during gestation, particularly in the early stages of development. TLRs are expressed by fetal and neonatal immune cells, while NK cells, macrophages, and DCs exhibit effector functions comparable to those observed in adults (229, 230). Furthermore, studies of Zika virus infection have demonstrated that the fetus can activate type I interferon-dependent antiviral pathways, thereby inducing the expression of genes involved in defense against viral infections (231). Maternal antibody transfer also plays a critical role in this context, becoming progressively more efficient throughout gestation and contributing to the newborn’s immunological protection (103).

Additionally, distinct fetal tissues actively participate in innate immunity. The skin, lungs, and gastrointestinal tract possess local mechanisms for pathogen recognition and the production of inflammatory mediators. Under intrauterine inflammatory conditions, such as chorioamnionitis, increased expression of immune receptors, cytokines, and chemokines has been observed, along with activation of resident immune cells. These changes promote the maturation of tissue barriers and the establishment of fetal immunity before birth (225).

Collectively, these findings indicate that maternal exposures during pregnancy can influence fetal immune development through multiple, and not necessarily mutually exclusive, pathways, including placental transfer of inflammatory mediators and microbial components, modulation of fetal hematopoietic and immune cells, and epigenetic, transcriptional, and metabolic reprogramming. The persistence and clinical significance of these effects vary considerably across experimental models and human studies, ranging from fetal or neonatal immune alterations to sustained changes in immune responsiveness and documented health outcomes later in life. **Table 1** integrates the available evidence linking maternal exposures to fetal immune targets, underlying mechanisms, persistent immune or developmental phenotypes, subsequent health outcomes, and the corresponding level of evidence.

**Table 1:** Maternal exposures and their potential impact on fetal immune programming and postnatal health outcomes.

Maternal exposure	Fetal immune target	Identified/proposed mechanism	Persistent immune/developmental phenotype	Documented postnatal/long-term outcome	Level of evidence	Reference(s)
Maternal exposure to a farming environment during pregnancy	Fetal/neonatal innate immune system	Prenatal exposure to a microbe-rich environment associated with modulation of innate immune receptor expression and immune maturation	Altered innate immune phenotype at birth, including increased expression of innate immune receptors	Reduced risk of allergic sensitization/atopic outcomes in childhood	Human – prospective birth cohort	(202)
Maternal antibiotic use during pregnancy	Developing fetal immune system/microbiome–immune axis	Prenatal antibiotic exposure, potentially through perturbation of maternal microbial communities and fetal/neonatal immune development	Not directly assessed	Increased risk of childhood asthma	Human – observational epidemiological evidence	(203)
Maternal antibiotic use during pregnancy	Developing fetal immune system/microbiome–immune axis	Prenatal antibiotic exposure associated with perturbation of early microbial and immune development	Not directly assessed	Increased risk of very early-onset inflammatory bowel disease/Crohn’s disease in offspring	Human – population-based epidemiological evidence	(204)
Maternal immune activation (MIA)/prenatal inflammation	Fetal HSCs and HSPCs	Inflammatory transcriptional activation of fetal HSC populations, promoting expansion of lymphoid-biased progenitors	Persistent expansion of fetal lymphoid-biased progenitors and hyperresponsiveness of fetal-derived innate-like lymphocytes after birth	Persistent alterations in postnatal hematopoiesis and immune function	Animal – experimental (mouse)	(209)
Maternal immune activation (MIA) during pregnancy	Developing fetal microglia	Persistent transcriptional, chromatin, and immunometabolic reprogramming of microglia	Long-lasting reduction in microglial immune reactivity	Alterations in neuronal circuitry and brain function in adult offspring	Animal – experimental/mechanistic (mouse)	(210)
Maternal immune activation (MIA)	Developing fetal neuroimmune landscape/neural progenitor cells	Sex-specific remodeling of immune ligand–receptor networks, including disruption of CXCL12/CXCR7 signaling	Altered fetal neurogenesis and neural progenitor development	Abnormal social and anxiety-related behaviors in adult male offspring	Animal – experimental (mouse)	(211)
Maternal microbiome depletion (MMD)	Developing fetal neuroimmune landscape/neural progenitor cells	Sex-specific remodeling of neuroimmune signaling networks, including CXCL12/CXCR7 alterations	Altered fetal neurogenesis and neural progenitor development	Abnormal social and anxiety-related behaviors in adult male offspring	Animal – experimental (mouse)	(211)
Maternal immune activation (MIA)	Developing fetal brain/neural and neuroimmune cell populations	Altered m6A RNA methylation and dysregulation of m6A regulatory machinery, particularly FTO	Persistent epitranscriptomic/transcriptional alterations affecting neurodevelopmental programs	Neurobehavioral abnormalities in adult offspring; modulation of FTO partially rescued MIA-associated behavioral phenotypes	Animal – experimental/mechanistic (mouse)	(212)
Maternal immune activation (MIA)	Developing fetal brain/IL-17RA-expressing cortical cells	Maternal TH17–IL-17A signaling induces IL-17RA-dependent alterations in fetal cortical development	Persistent abnormalities in cortical development/architecture	Autism-like behavioral phenotypes in offspring	Animal – experimental/mechanistic (mouse)	(214)
Maternal HIV-1 infection without vertical transmission	Fetal/neonatal CD4+ T cells and innate immune compartment	*In utero* immune priming associated with maternal/placental inflammation, altered chemokine receptor expression, altered transplacental immune factor transfer, and modified responsiveness to PAMPs	Altered CD4+ T-cell activation/homing and enhanced pro-inflammatory innate responses during early infancy	Increased susceptibility to infectious morbidity in HIV-exposed uninfected infants	Human – cord blood/ex vivo, prospective longitudinal, and clinical evidence	(213, 215, 216)
Maternal/placental *Plasmodium falciparum* infection during pregnancy	Fetal/neonatal innate immune cells and TLR signaling pathways	*In utero* immune conditioning associated with altered TLR3-, TLR7/8-, and TLR9-mediated cytokine responses	Altered innate cytokine responsiveness from birth through infancy, with exposure-dependent inflammatory/regulatory profiles	Differential susceptibility to *P. falciparum* infection and clinical malaria during the first year of life	Human – prospective longitudinal birth cohorts	(217, 218)
Maternal chronic HBV infection/fetal HBV exposure *in utero*	Fetal/neonatal monocytes and innate immune cells; developing Th1 compartment	Innate immune maturation and transcriptional reprogramming associated with reduced IL-10 and increased IL-12p40 and IFN-α2	Trained innate immune phenotype and enhanced Th1 development detectable in cord blood	Enhanced responsiveness of neonatal immune cells to unrelated bacterial pathogens *in vitro*; long-term clinical outcomes not assessed	Human – cord blood/ex vivo mechanistic study	(219)
Maternal history of BCG vaccination (BCG scar)	Fetal/neonatal innate immune system	Maternal BCG-associated modulation of fetal innate immune responsiveness; transplacental mechanism not directly established	Enhanced pro-inflammatory responses in cord blood and upregulated interferon/inflammatory pathways following neonatal BCG vaccination	Altered early-life immune response to neonatal BCG vaccination; later clinical outcomes not assessed	Human – mother–infant observational study	(220)
Maternal IL-6 exposure during pregnancy	Developing fetal compartment	Direct maternal-to-fetal transfer of IL-6, with substantially greater transfer during mid-gestation than late gestation	Not assessed	Postnatal outcome not assessed	Animal – experimental (rat)	(221)
Maternal inflammatory cytokine exposure (IL-6)	Fetal compartment across the term placenta	Bidirectional transplacental transfer of IL-6 was reported, whereas TNF-α and IL-1α showed minimal transfer	Not assessed	Postnatal outcome not assessed	Human – ex vivo term placental perfusion	(130)
Maternal inflammatory cytokine exposure (IL-6, TNF-α, IL-1)	Fetal compartment across the term placenta	No detectable transplacental transfer of IL-6, TNF-α, or IL-1 in either direction across normal term placenta	Not assessed	Postnatal outcome not assessed	Human – ex vivo term placental perfusion	(129)
Maternal G-CSF administration during pregnancy	Fetal hematopoietic/myeloid compartment	Transplacental transfer of maternally administered G-CSF, stimulating fetal granulopoiesis and expanding neutrophil pools	Increased neonatal circulating neutrophils and expansion of myeloid/neutrophil pools in bone marrow and spleen	Markedly increased neonatal survival following lethal Group B *Streptococcus* challenge	Animal – experimental (rat)	(222, 223)
Maternal exposure to bacterial peptidoglycan (PGN) during pregnancy	Developing fetal brain/neural progenitor cells	Transplacental passage of bacterial PGN triggering fetal neuroproliferative signaling	Altered fetal neurodevelopment following increased neuroproliferation	Aberrant postnatal behavior	Animal – experimental	(224)
Choriodecidual Group B *Streptococcus* (GBS) exposure during pregnancy	Fetal lung and fetal inflammatory compartment	Choriodecidual infection induces inflammatory cytokine signaling and fetal inflammation despite absence of detectable intra-amniotic or fetal GBS infection	Fetal lung injury characterized by intra-alveolar neutrophils and interstitial thickening	Postnatal outcome not assessed	Animal – experimental (nonhuman primate)	(226)

The table summarizes evidence linking maternal exposures during pregnancy to fetal immune or developmental targets, the underlying mechanisms involved, persistent immune or developmental phenotypes, and documented postnatal or long-term outcomes. The level and type of evidence are indicated for each study, including human observational, ex vivo, and animal experimental evidence. Where persistent phenotypes or postnatal outcomes were not evaluated, these are indicated as “not assessed”.

## 2 Concluding remarks and future perspectives

The contemporary understanding of the fetal and neonatal immune system has shifted from a paradigm of “intrinsic immaturity” to one of “developmental specialization.” As discussed throughout this review, the prenatal period is not a static phase of immunological dormancy, but a highly dynamic epoch characterized by complex hematopoietic choreography, the establishment of maternal-fetal tolerance, and a unique form of kinetic plasticity. This system is actively “calibrated” by environmental, microbial, and maternal cues, laying the foundation for the individual’s definitive immunological landscape.

The phenomenon of immune programming, mediated by metabolic and epigenetic shifts, defines the threshold for responsiveness to future challenges. The transition from an “expansion-driven” fetal state to a “homeostasis-driven” adult system is not merely an anatomical relocation but a fundamental evolutionary shift in the regulation of genomic integrity versus effector adaptability. Central to this transition is trained immunity, an antigen-agnostic memory that allows the innate immune system to respond with heightened vigor to heterologous threats, effectively bridging the gap between prenatal development and postnatal survival.

For the field of immunogenomics and precision immunotherapy, these insights present both a challenge and a revolutionary opportunity. We propose that the early-life immunological *imprint*, the culmination of fetal programming and microbial education, acts as a significant yet underinvestigated variable in determining clinical outcomes. Identifying the specific immunogenomic biomarkers resulting from these early developmental windows could potentially allow us to:

1. Stratify patients based on their “immunological history” to predict their baseline capacity to respond to inflammatory stimuli or immunotherapeutic agents.
2. Refine neonatal clinical practices, specifically the management of early life dysbiosis and antimicrobial use, to preserve the “functional readiness” of the innate compartment.
3. Optimize immunotherapeutic strategies for adults by accounting for developmental constraints, potentially leveraging epigenetic “priming” to enhance treatment efficacy.

Ultimately, shifting our focus toward the early-life programming of the immune system represents a paradigm shift in precision medicine. Future research must prioritize the longitudinal mapping of these developmental trajectories, moving toward a holistic view in which the origins of adult immune competence are understood to be deeply intertwined with the molecular dialogue occurring at the maternal-fetal interface. Deciphering this code will be essential for the next generation of patient-centered immunotherapies, ensuring that we no longer treat the immune system as a static entity, but as a dynamic history of the individual’s earliest environmental encounters.
